# Supplementary material for: p38-MK2 signaling axis regulates RNA metabolism after UV-light-induced DNA damage
Source: Nat Commun. 2018 Mar 9;9:1017. doi: 10.1038/s41467-018-03417-3 (PMC5845016; doi:10.1038/s41467-018-03417-3)
Supplement: Supplementary file 1 — Supplementary Information [file 41467_2018_3417_MOESM1_ESM.pdf]

p38-MK2 signaling axis regulates RNA metabolism after UV light-induced DNA damage

Borisova et al.

Supplementary Figure 1

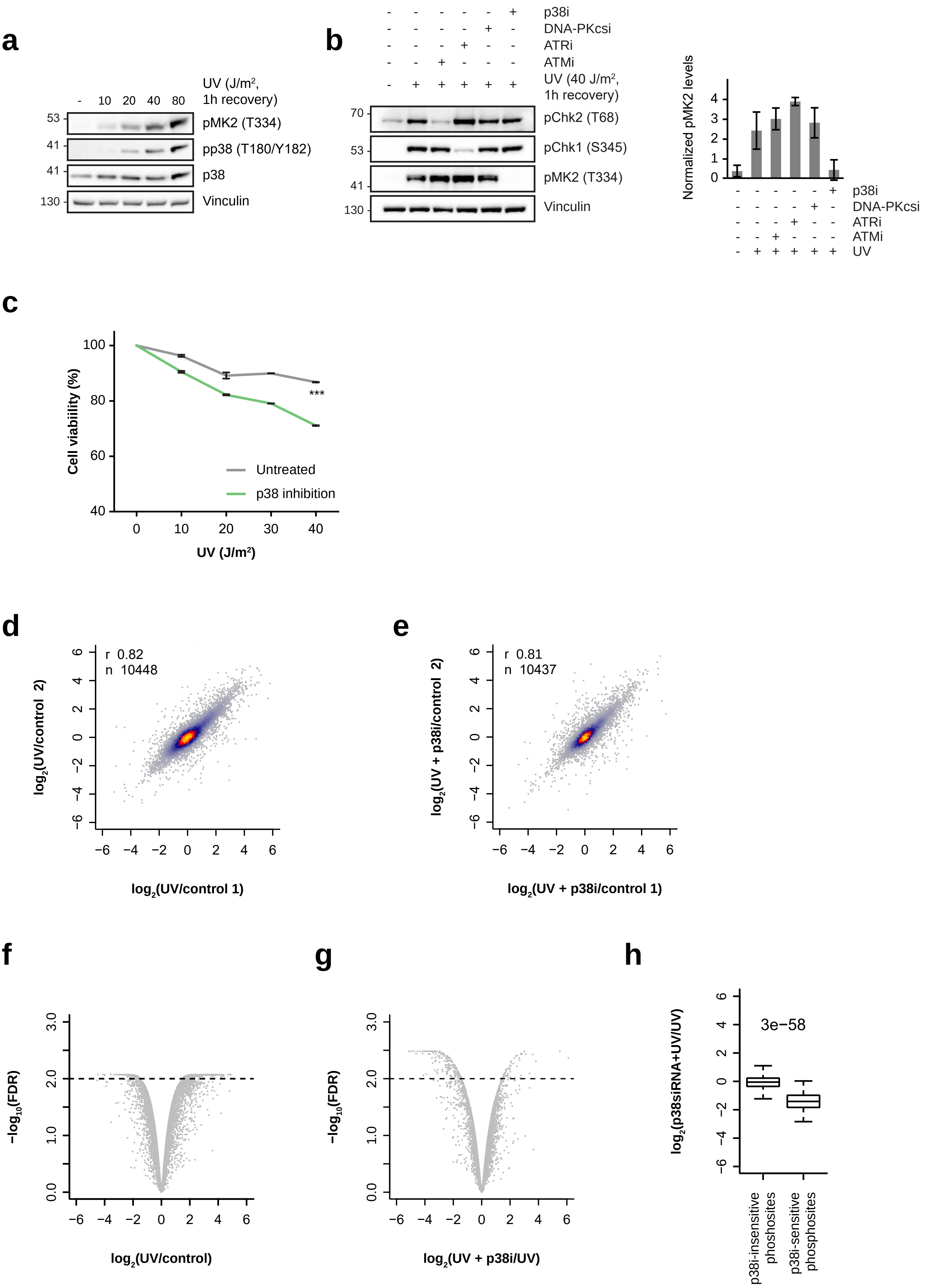

Supplementary Figure 1: Proteome-wide identification of p38-dependent phosphorylation sites

a. U2OS cells were treated with increasing doses of UV light (10 - 80 J/m^2) and left to recover for 1 hour. Total cell lysates were resolved on SDS-PAGE and activation of p38 was monitored with phospho-specific antibodies.

b. U2OS cells were pretreated for 1 hour with ATM inhibitor (KU-55933, 10 µM), ATR inhibitor (VE-821, 1 µM), DNA-PKcs inhibitor (KU-57788, 10 µM) or p38 inhibitor (SB 203580, 10 µM) and then irradiated with UV light (40 J/m^2, 1 hour recovery). Total cell lysates were resolved on SDS-PAGE and blotted with the indicated antibodies (left). The bar plot shows the mean and standard deviation of normalized pMK2 levels quantified from three replicate experiments (right).

c. Cell viability was measured for mock-treated U2OS cells and cells irradiated with different doses of UV light without and with 1 hour pretreatment with the p38 inhibitor. The plot shows the mean and standard deviation of the results obtained in three biological replicate experiments, each performed in three technical replicates. Two-sided Student's t-test was used to assess the significance (*** p value < 0.001).

d. Scatter plot shows the logarithmized SILAC ratios UV/control of quantified phosphorylation sites in replicate experiments. The color-coding indicates the density. The Spearman's rank correlation was calculated to determine the experimental reproducibility.

e. Scatter plot shows the logarithmized SILAC ratios UV + p38i/control of quantified phosphorylation sites in replicate experiments.

f. Identification of significantly regulated phosphorylation sites after p38 inhibition from two replicate experiments using the limma algorithm. P value < 0.01 was used as cut-off to determine phosphorylation sites that significantly increase after UV light.

g. Identification of significantly regulated phosphorylation sites after p38 inhibition from two replicate experiments was done using the limma algorithm. P value < 0.01 was used as cut-off to determine downregulated phosphorylation sites after p38 inhibition.

h. The box plot shows the SILAC ratio of p38i-insensitive sites and p38i-sensitive sites quantified after transient knockdown of p38. Box plot represents the 25th to 75th quartiles with the horizontal line representing the median value.

Supplementary Figure 2

a

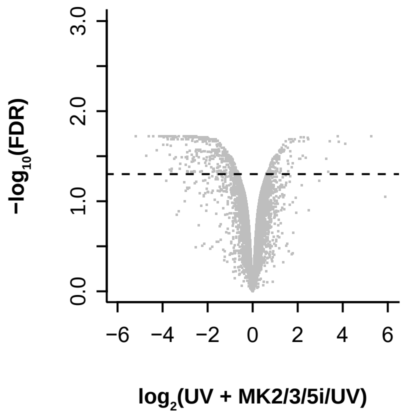

b

SILAC	Light Arg^0/Lys^0	Medium Arg^6/Lys^4	Heavy Arg^{10}/Lys^8
Transfection	Empty vector	Flag-Strep 14-3-3	Flag-Strep 14-3-3
Inhibitor	DMSO	DMSO	p38
UV	+	+	+

Supplementary Figure 2: Phosphorylation by p38-MK2 induces 14-3-3 binding to cellular proteins

a. Identification of significantly regulated phosphorylation sites after MK2/3/5 inhibition from two replicate experiments was done using the limma algorithm. P value < 0.05 was used as cut-off to determine downregulated phosphorylation sites after p38 or MK2/3/5 inhibition.

b. Schematic representation of the strategy used to identify UV light-induced, p38-dependent 14-3-3 interaction partners. SILAC-labeled U2OS cells transfected with Flag-Strep-14-3-3 were mock-treated, irradiated with UV light (40 J/m^2, 1 hour recovery) or pretreated with the p38 inhibitor and irradiated with UV light. 14-3-3 and its binding partners were enriched using StrepTactin sepharose, digested in-gel into peptides and peptide samples were analyzed by LC-MS/MS.

Supplementary Figure 3

a

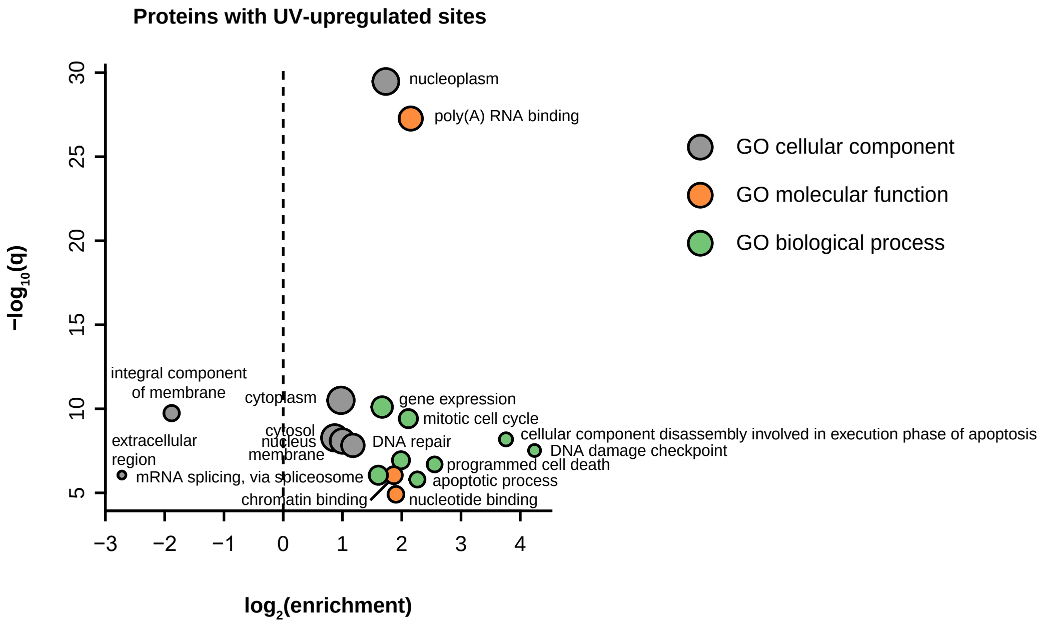

b

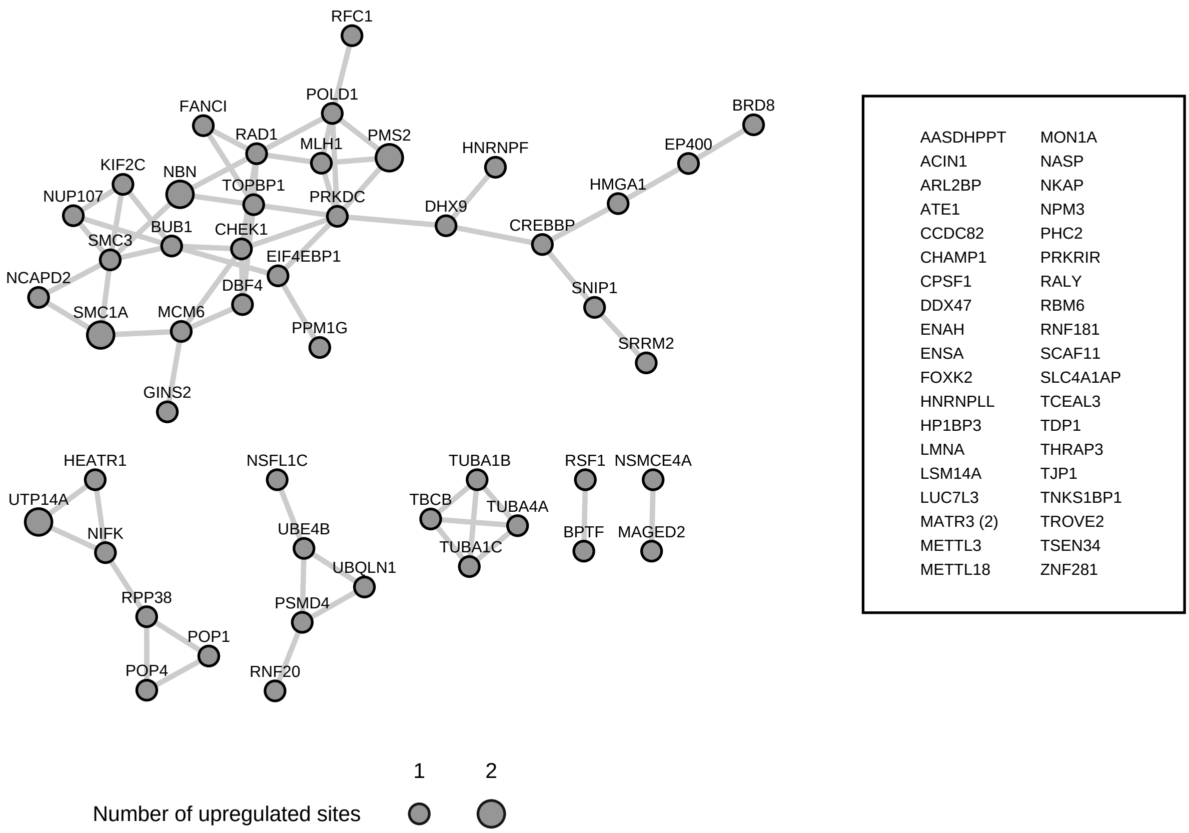

Supplementary Figure 3: Analysis of UV light-induced phosphorylation sites

a. GO terms significantly enriched among proteins with UV light-induced phosphorylation sites. The dot plot shows significantly overrepresented GO terms associated with proteins containing UV light-induced phosphorylation sites compared to proteins containing non-regulated sites. The significance of the enrichment of a specific term was determined using Fisher's exact test. P values were corrected for multiple hypotheses testing using the Benjamini and Hochberg FDR.

b. Analysis of functional associations among proteins with UV light-induced S/TQ phosphorylation sites. Functional interactions were obtained from the STRING database and visualized using Cytoscape. Proteins with S/TQ sites not involved in functional interactions are indicated on the right.

Supplementary Figure 4

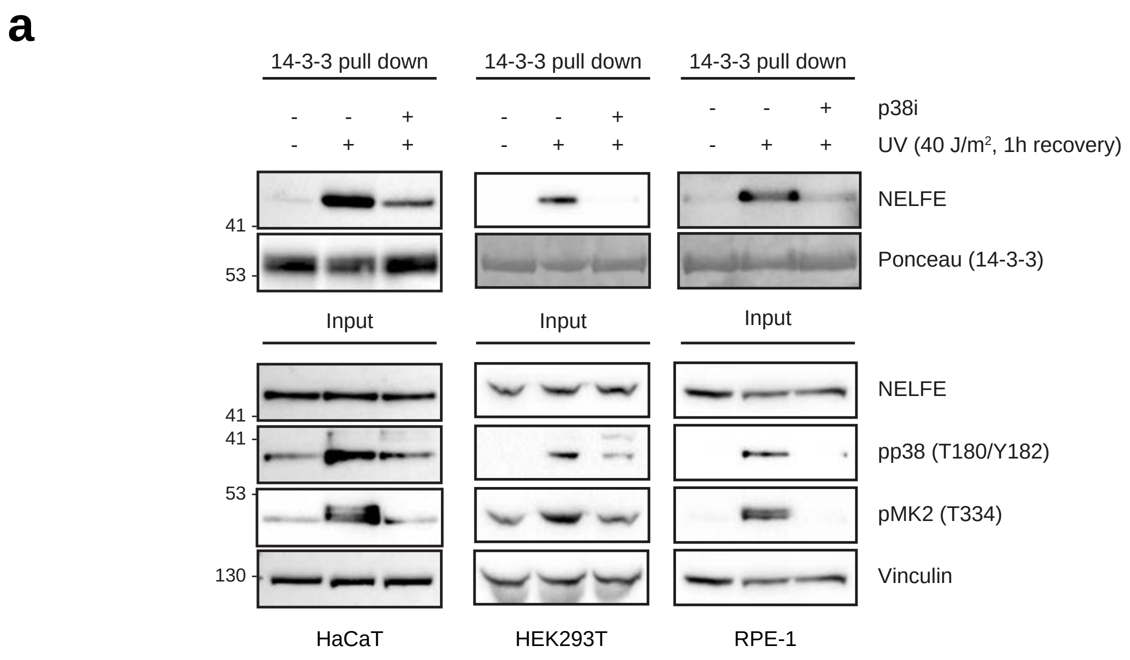

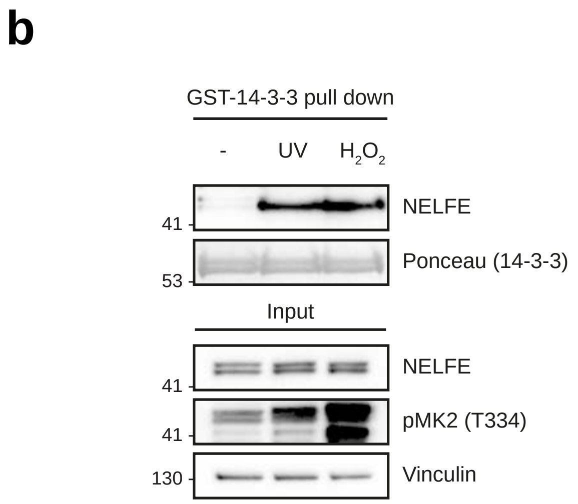

Supplementary Figure 4: MK2-dependent phosphorylation of NELFE promotes its binding to 14-3-3

a. Validation of interaction between NELFE and 14-3-3 in HaCaT, HEK293T and RPE-1 cells. Total cell extracts from differentially treated cells were incubated with the recombinant GST-14-3-3. Enriched proteins were resolved by SDS-PAGE and subjected to western blotting.
b. NELFE interacts with 14-3-3 after oxidative stress induced by treatment with H_2O_2 for 1 hour. Total cell extracts from differentially treated U2OS cells were incubated with the recombinant GST-14-3-3. Enriched proteins were resolved by SDS-PAGE and subjected to western blotting.

Supplementary Figure 5

a

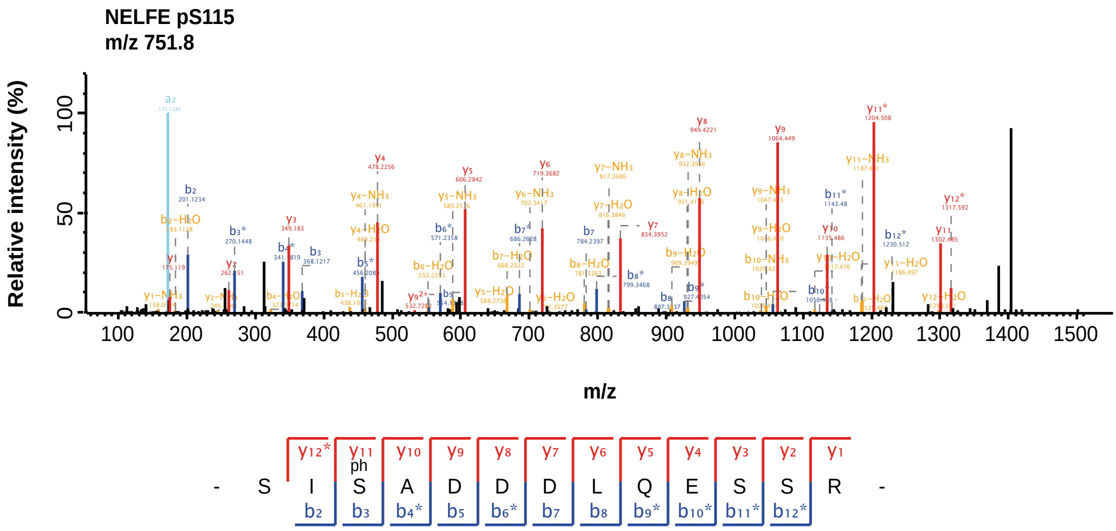

b

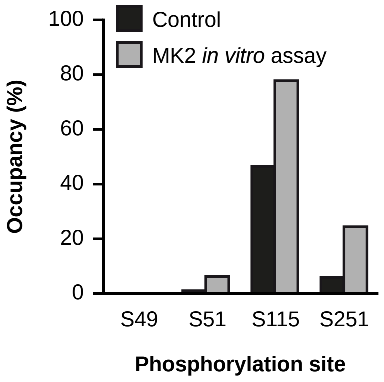

c

NELFE (95-140 aa)

H. sapiens	100	EKGPVPTFQPFQR---SISADDDLQ-ESSRRPQRKSLYESF	136
M. mulatta	100	EKGPVPTFQPFQR---SISADDDLQ-ESSRRPQRKSLYESF	136
P. troglodytes	100	EKGPVPTFQPFQR---SISADDDLQ-ESSRHPQRKSLYESF	136
F. catus	100	EKGPVPTFQPFQR---SISADDDLQ-ESSRRPQRKSLYESF	136
B. taurus	100	EKGPVPTFQPFQR---SVSADDDLQ-ESSRRPQRKSLYESF	136
R. norvegicus	118	EKGPVPTFQPFQR---SMSADEDLQ-EPSRRPQRKSLYESF	154
M. musculus	100	EKGPVPTFQPFQR---SMSADEDLQ-EPSRRPQRKSLYESF	136
M. domestica	100	EKGPAPTFQPFQR---SISADDDLQ-ESSRRPQRKSLYESF	136
T. rubripes	100	EKGPVPAFLPFQR---SVSADDE-P-ESAKRVHRKSLYESF	135
D. rerio	100	EKGPAPAFLPFQR---SVSTDEE-PpDSAKRIHRKSLYESF	136
X. tropicalis	100	DKGPVPSFQPFQR---SVSVDEE-QaESSRRSQRKSLYESF	136
D. melanogaster	96	SETTVASYQPFsstQNDVAQETIISeIIKEEPRRQNLYQHF	136

d

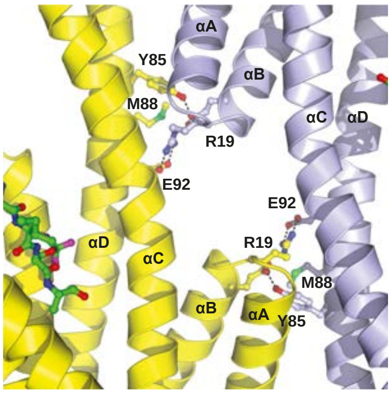

e

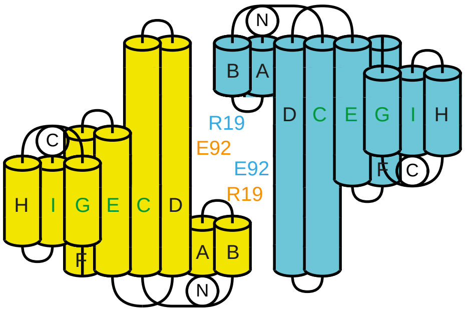

Supplementary Figure 5: NELFE phosphorylation by MK2 promotes its binding to 14-3-3

a. Mass spectrometric fragment ion scan of the peptide corresponding to phosphorylated serine 115 in NELFE.

b. Purified MK2 can phosphorylate immunoprecipitated NELFE on S51, S115 and S251 *in vitro*.

c. Conservation of the NELFE peptide sequence corresponding to serine 115 across evolution.

d. Structure of the 14-3-3 epsilon homo dimer in cartoon representation (Yellow and Cyan).

e. Topology diagrams of the 14-3-3 epsilon. Topology diagrams were prepared with TopDraw.

Supplementary Figure 6

a

SILAC	Light Arg^0/Lys^0	Medium Arg^6/Lys^4	Heavy Arg^{10}/Lys^8
Inhibitor	DMSO	DMSO	p38
UV		+	+

↓

Chromatin proteome analysis

b

	Ratio M/L 1	Ratio H/L 1	Ratio H/M 1	Ratio M/L 2	Ratio H/L 2	Ratio H/M 2
Ratio M/L 2	0.71			/		
Ratio H/L 2		0.6			/	
Ratio H/M 2			0.54			/
Ratio M/L 3	0.64			0.68		
Ratio H/L 3		0.59			0.62	
Ratio H/M 3			0.51			0.58

c

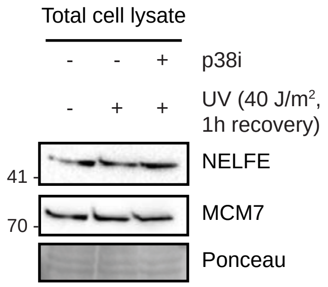

d

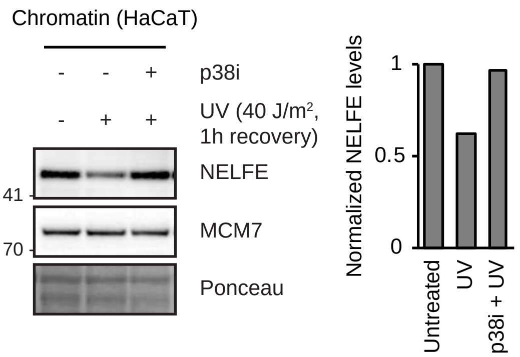

e

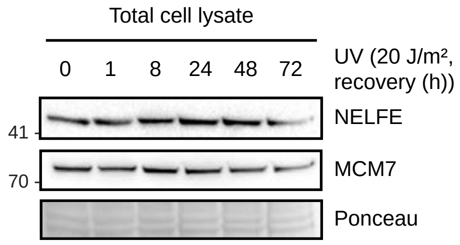

Supplementary Figure 6: Protein dynamics on chromatin after UV light

a. Schematic representation of the strategy used to identify UV light-induced, p38-dependent change in the chromatin proteome. SILAC-labeled U2OS cells were mock-treated, irradiated with UV light (40 J/m^2, 1 hour recovery) or pretreated with the p38 inhibitor and irradiated with UV light. Chromatin-associated proteins were extracted from cells, digested in-gel into peptides and peptide samples were analyzed by LC-MS/MS.
b. The Spearman's rank correlation was calculated to determine the experimental reproducibility.
c. Total cell lysates of U2OS cells were resolved by SDS-PAGE and proteins were detected with the indicated antibodies.
d. NELFE dissociates from chromatin in a p38-dependent manner after UV light in HaCaT cells. Chromatin protein fractions from differentially treated cells were resolved by SDS-PAGE and subjected to western blotting with the indicated antibodies.
e. U2OS cells were exposed to UV light and left to recover for different time points. Total cell lysates were resolved by SDS-PAGE and proteins were detected with the indicated antibodies.

Supplementary Figure 7

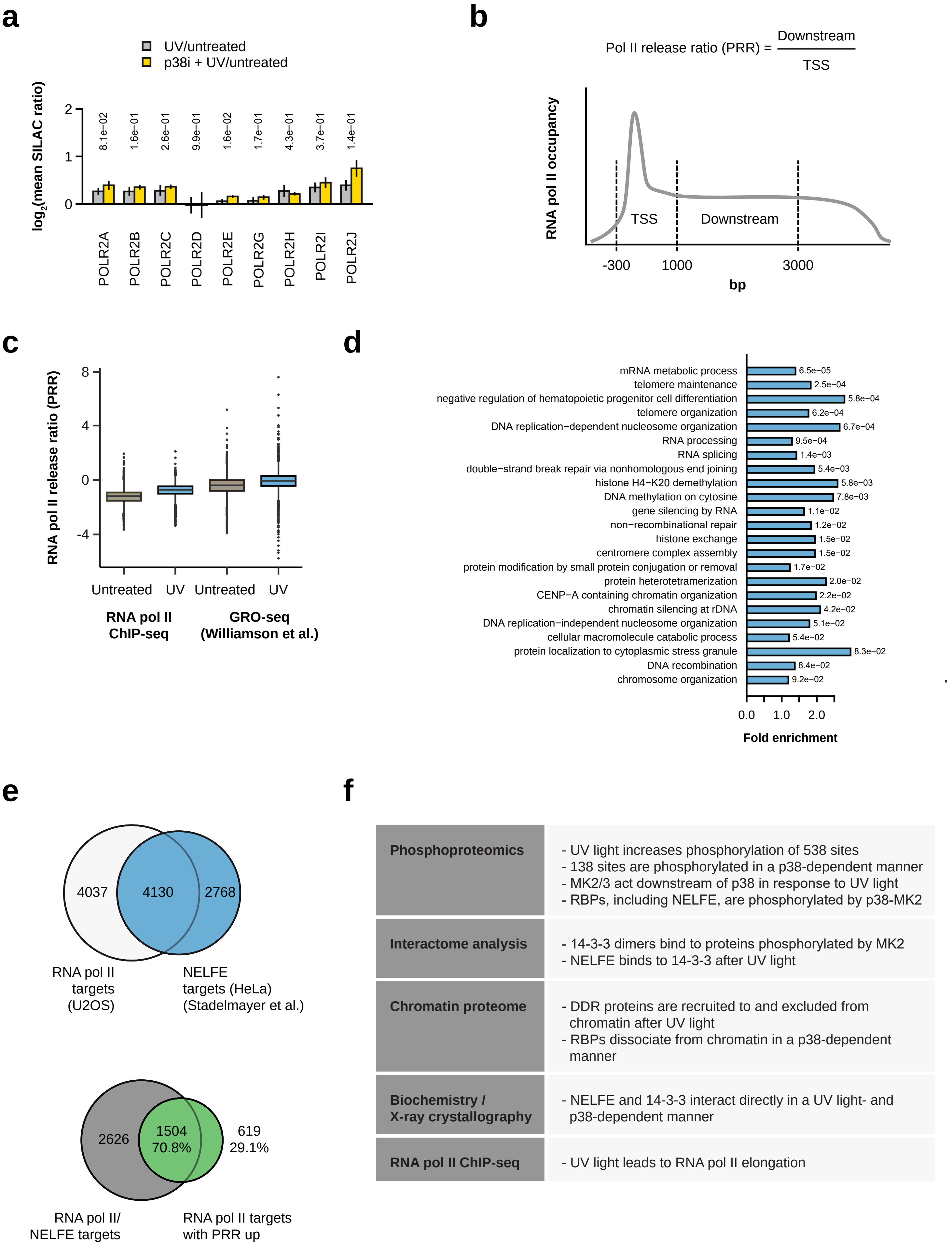

Supplementary Figure 7: UV light exposure leads to transcriptional elongation

a. The bar plot shows the levels of RNA pol II subunits on chromatin quantified in untreated U2OS cells and after UV light by SILAC-based quantitative MS. The error bars show the mean and standard deviation of SILAC ratios quantified from three replicate experiments. Two sided Student's t test was used to assess the significance.
b. Schematic representation of the approach for the RNA pol II release ratio (PRR) calculation.
c. Comparison of PRRs calculated in untreated and UV light treated U2OS cells from two RNA pol II ChiP-seq replicate experiments with PRRs calculated from GRO-seq data from the study by Williamson et al. The lower and upper hinges represent the first and third quartiles (25th and 75th percentiles, respectively). The line in the center of the box corresponds to the median of the data range.
d. GO terms significantly enriched among genes with UV light-upregulated PRR. The bar plot shows significantly overrepresented GO terms associated with genes containing upregulated PRR compared to all RNA pol II bound genes. The significance of the enrichment of a specific term was determined using a hypergeometric test. P values were corrected for multiple hypotheses testing using the Benjamini and Hochberg FDR.
e. NELFE target genes were extracted from the study by Stadelmayer et al. and overlapped with genes displaying significantly increased PRRs after UV light exposure determined by RNA pol II ChiP-seq. 70.8% of genes with increased PRR were found to be targets of NELFE in HeLa cells.
f. A schematic overview of the methods and findings reported in this study.

Supplementary Figure 8

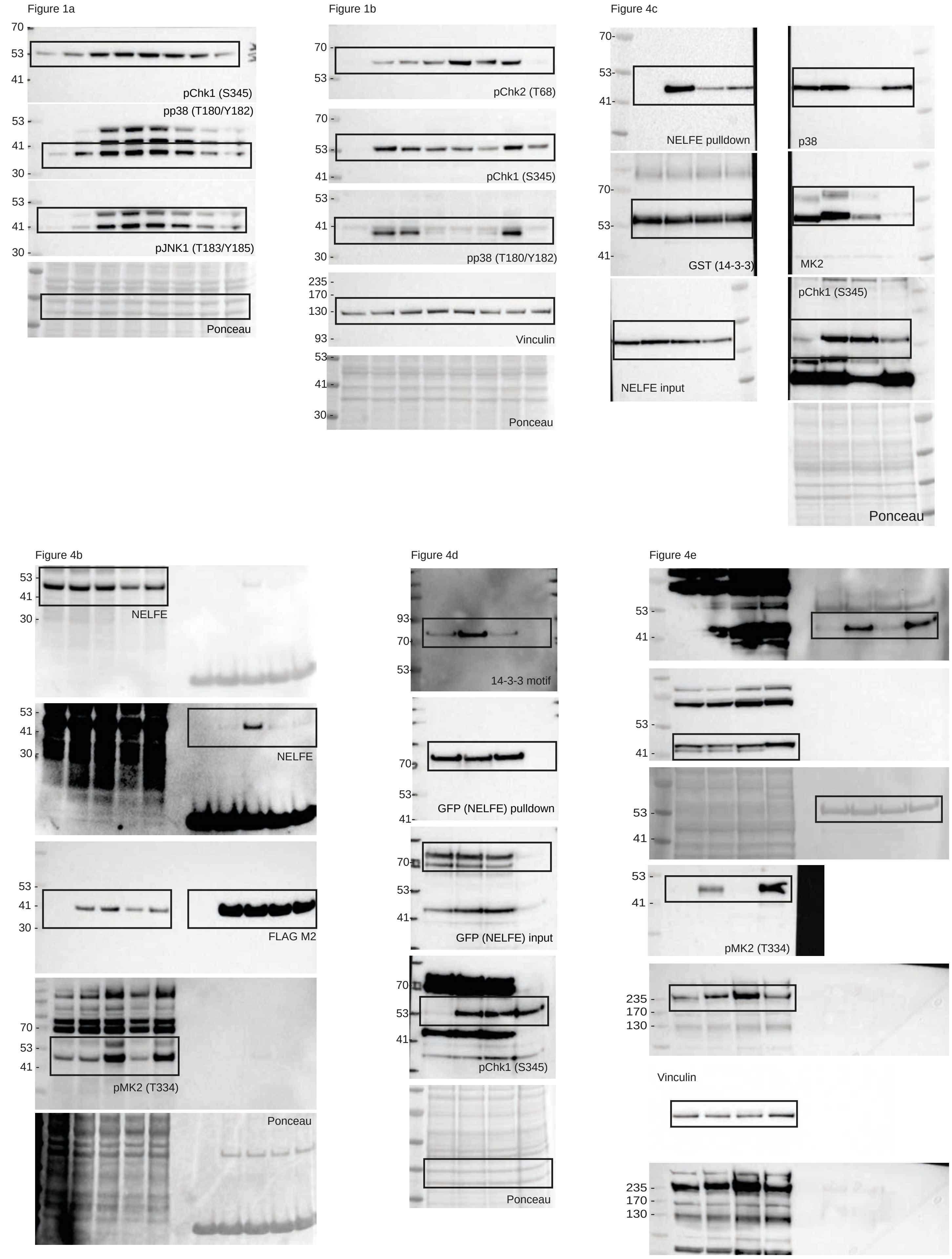

Figure 4f

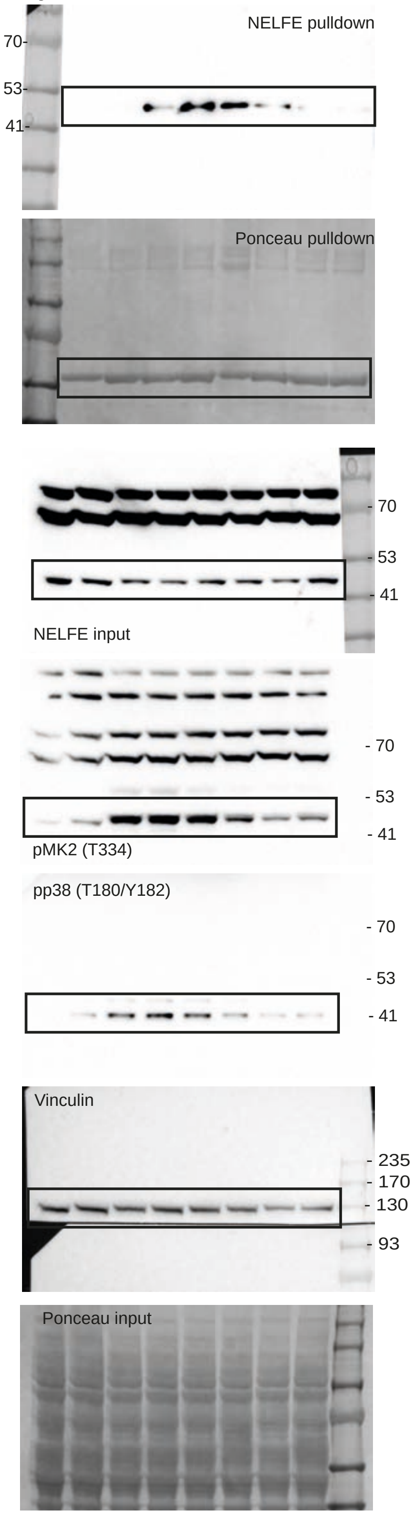

Figure 4g

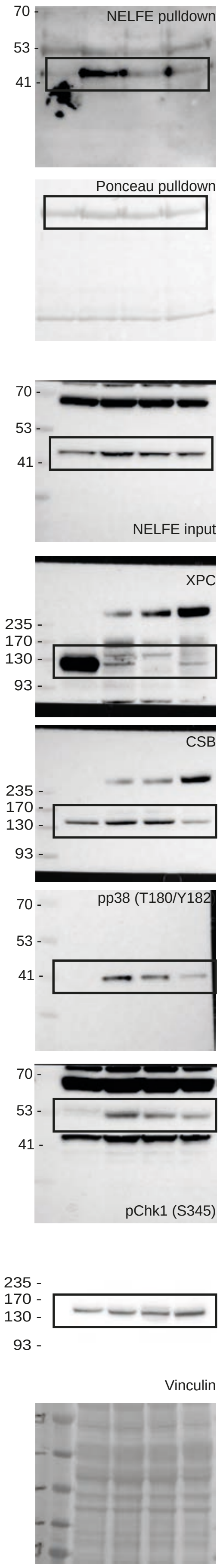

Figure 5c

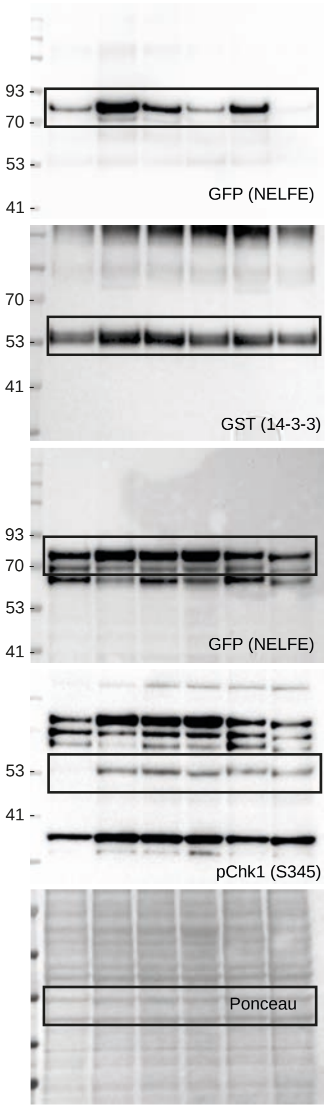

Figure 5g

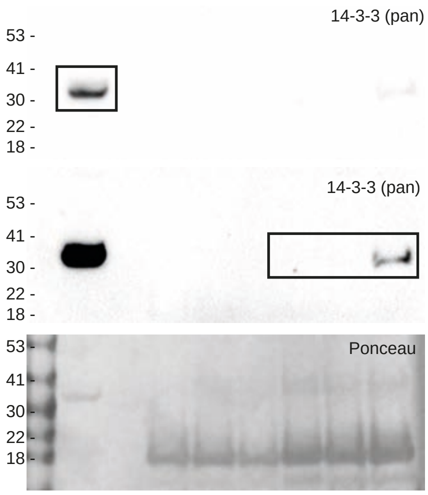

Figure 6d

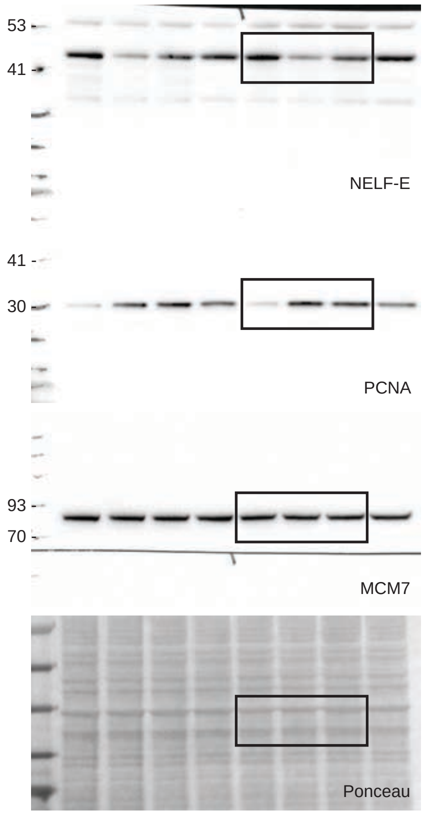

Figure 6e

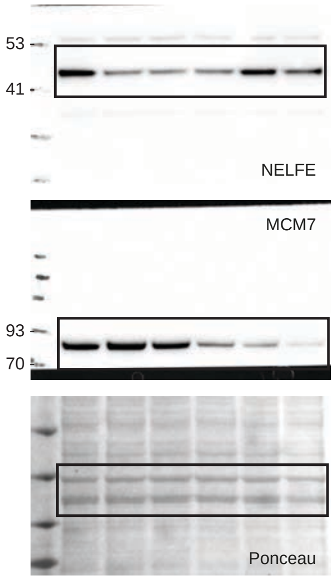

Figure S1a

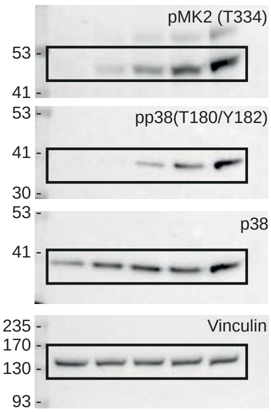

Figure S1b

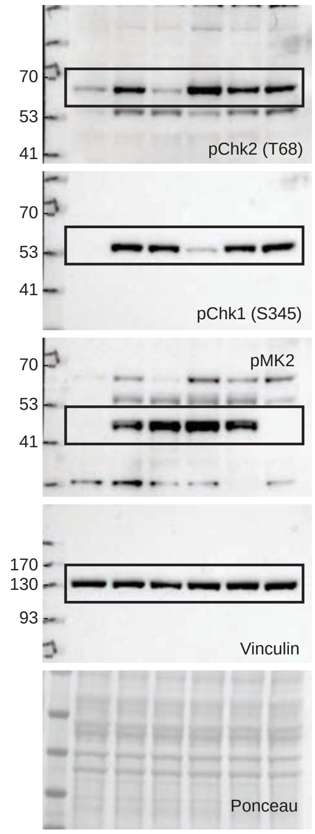

Figure S4a

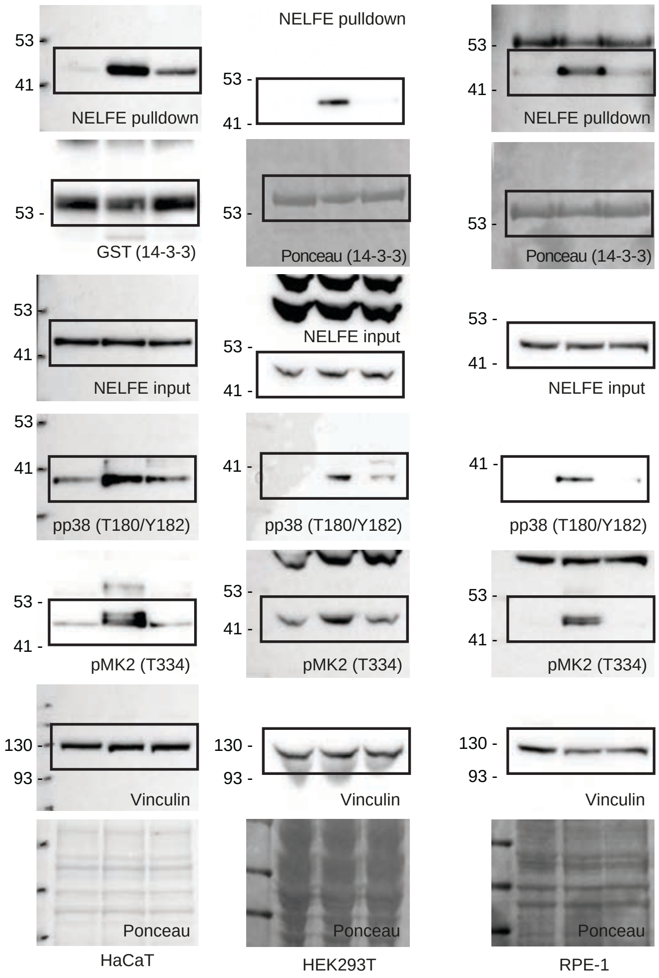

Figure S4b

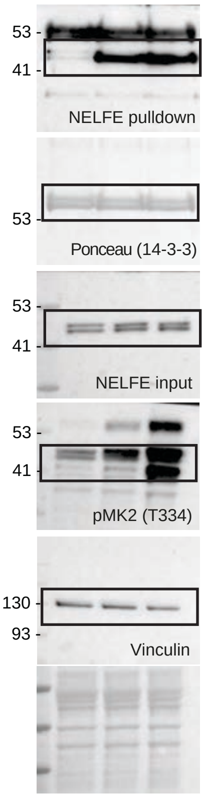

Figure S6c and S6d

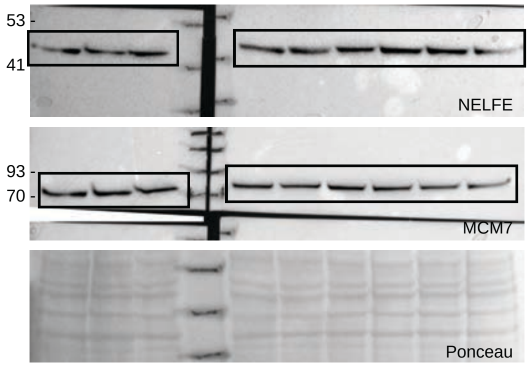

Figure S6e

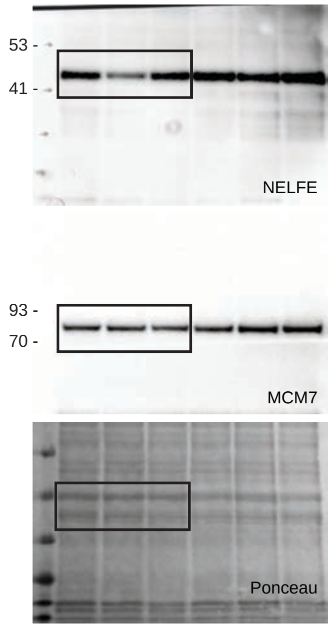

Supplementary Figure 8: Uncropped scans of all western blots

Supplementary Table 1

List of siRNAs used in this study

Gene name	Sequence 5’-3’
p38	gaagcucuccagaccauuu
MK2	ccgaaaucaugaagagcau
MK3	ccaaagauguugugaggaa
MK5	ggagaaagacgcagugcuu
NELFE 1	cagccaagguggugucaaa
NELFE 3’UTR	acacugagguggaagcuuac
XPC	gcaaauggcuucuaucgaa
CSB	ccactgattacgagataca

Supplementary Table 2

List of oligos used in this study

Construct	Sequence 5’-3’
NELFE S49/51A	gacaggctgctctgctagtgcgcgtttgacaccac
NELFE S115A	gtcatcatcagcagctatgctcctctggaacgg
NELFE S251A	ccgttcagggaatgcatccgacctgcgga

Supplementary Table 3

Crystallization data collection and refinement statistics. Values in parentheses are for the highest resolution shell.

	14-3-3/NELFE
**Data collection statistics**	
Beamline	SLS PX III
Wavelength (Å)	1.000
Space Group	$C\,2\,2\,2_1$
Unit Cell (Å)	a = 79.29, b = 81.19, c = 81.05<br>α =90.00, β = 90.00, γ = 90.00
Resolution (Å)	46.47 - 2.70<br>(2.85 - 2.70)
Observed reflections	97555 (14510)
Unique reflections	7463 (1059)
Redundancy	13.1 (13.7)
Completeness (%)	100.0 (100.0)
R_{merge}	0.126 (0.821)
$\langle I/\sigma I \rangle$	18.4 (3.5)
**Refinement statistics**	
Reflections in test set	757
R_{cryst}	17.2
R_{free}	24.1
**Number of groups**	
Protein residues	234
Ions and ligand atoms	0
Water	4
Wilson B-factor	50.55
**RMSD from ideal geometry**	
Bond length (Å)	0.012
Bond angles (°)	1.493
**Ramachandran Plot Statistics**	
In Favoured Regions (%)	224 (97.39)
In Allowed Regions (%)	6 (2.61)
Outliers (%)	0 (0.00)

Supplementary Table 4

List of antibodies used in this study

Protein name	Product number	Origin	Dilution
GFP	sc-9996	Santa Cruz	1:2000
FLAG	F1804	Sigma	1:2000
pp38 (T180/Y182)	9216	CST	1:1000
p38	8690	CST	1:1000
pMK2 (T334)	3007	CST	1:1000
pJNK	9255	CST	1:1000
pCHEK2 (T68)	2661	CST	1:1000
pCHEK1 (S345)	2344	CST	1:1000
Vinculin	V9264	Sigma	1:1000
NELFE	ABE48	Millipore	1:1000
14-3-3 (pan)	8312	CST	1:1000
POLR2A	sc-899	Santa Cruz	ChIP
pCTD (S2)	13499	CST	1:1000
XPC	14768	CST	1:1000
CSB	sc-398022	Santa Cruz	1:1000
GST	sc-138	Santa Cruz	1:2000
PCNA	sc-56	Santa Cruz	1:1000
MCM7	3735	CST	1:1000
MCM6	sc-9843	Santa Cruz	1:1000
p14-3-3 motif	9601	CST	1:1000
