# Supplementary material for: p38-MK2 signaling axis regulates RNA metabolism after UV-light-induced DNA damage
Source: Nat Commun. 2018 Mar 9;9:1017. doi: 10.1038/s41467-018-03417-3 (PMC5845016; doi:10.1038/s41467-018-03417-3)
Supplement: Supplementary file 3 — Description of Additional Supplementary Files [file 41467_2018_3417_MOESM3_ESM.pdf]

Description of Additional Supplementary Files

File Name: Supplementary Data 1
Description: The excel workbook contains a spreadsheet with all quantified phosphorylated peptides from U2OS cells treated as described in Figure 1c.

File Name: Supplementary Data 2
Description: The excel workbook contains a spreadsheet with all quantified phosphorylated peptides from U2OS cells treated as described in Figure 2a.

File Name: Supplementary Data 3
Description: The excel workbook contains a spreadsheet with all quantified phosphorylated peptides from U2OS after knockdown of MK2/3 or MK5 (SILAC light: UV light, control siRNA; SILAC medium: UV light, MK2/3 knockdown; SILAC heavy: UV light, MK5 knockdown)

File Name: Supplementary Data 4
Description: The excel workbook contains a spreadsheet with all quantified protein groups in 14-3-3 pull downs from U2OS cells treated as described in Supplementary Figure 2b.

File Name: Supplementary Data 5
Description: The excel workbook contains a spreadsheet with all quantified chromatin-associated proteins from U2OS cells treated as described in Supplementary Figure 6a.

File Name: Supplementary Data 6
Description: The excel workbook contains a spreadsheet with calculated PRRs in mock-treated and UV light-exposed cells (40 J/m2, 1 hour recovery) from RNA pol II ChiP-seq data obtained in U2OS cells.
